# Computational Modeling of Fluid Flow and Intra-Ocular Pressure following Glaucoma Surgery

**DOI:** 10.1371/journal.pone.0013178

**Published:** 2010-10-04

**Authors:** Bruce S. Gardiner, David W. Smith, Michael Coote, Jonathan G. Crowston

**Affiliations:** 1 School of Computer Science and Software Engineering, The University of Western Australia, Crawley, Western Australia, Australia; 2 Centre for Eye Research Australia (CERA), The University of Melbourne and the Royal Victorian Eye and Ear Hospital, Melbourne, Victoria, Australia; University of Arizona, United States of America

## Abstract

**Background:**

Glaucoma surgery is the most effective means for lowering intraocular pressure by providing a new route for fluid to exit the eye. This new pathway is through the sclera of the eye into sub-conjunctival tissue, where a fluid filled bleb typically forms under the conjunctiva. The long-term success of the procedure relies on the capacity of the sub-conjunctival tissue to absorb the excess fluid presented to it, without generating excessive scar tissue during tissue remodeling that will shut-down fluid flow. The role of inflammatory factors that promote scarring are well researched yet little is known regarding the impact of physical forces on the healing response.

**Methodology:**

To help elucidate the interplay of physical factors controlling the distribution and absorption of aqueous humor in sub-conjunctival tissue, and tissue remodeling, we have developed a computational model of fluid production in the eye and removal via the trabecular/uveoscleral pathways and the surgical pathway. This surgical pathway is then linked to a porous media computational model of a fluid bleb positioned within the sub-conjunctival tissue. The computational analysis is centered on typical functioning bleb geometry found in a human eye following glaucoma surgery. A parametric study is conducted of changes in fluid absorption by the sub-conjunctival blood vessels, changes in hydraulic conductivity due to scarring, and changes in bleb size and shape, and eye outflow facility.

**Conclusions:**

This study is motivated by the fact that some blebs are known to have ‘successful’ characteristics that are generally described by clinicians as being low, diffuse and large without the formation of a distinct sub-conjunctival encapsulation. The model predictions are shown to accord with clinical observations in a number of key ways, specifically the variation of intra-ocular pressure with bleb size and shape and the correspondence between sites of predicted maximum interstitial fluid pressure and key features observed in blebs, which gives validity to the model described here. This model should contribute to a more complete explanation of the physical processes behind successful bleb characteristics and provide a new basis for clinically grading blebs.

## Introduction

**Figure 1:**
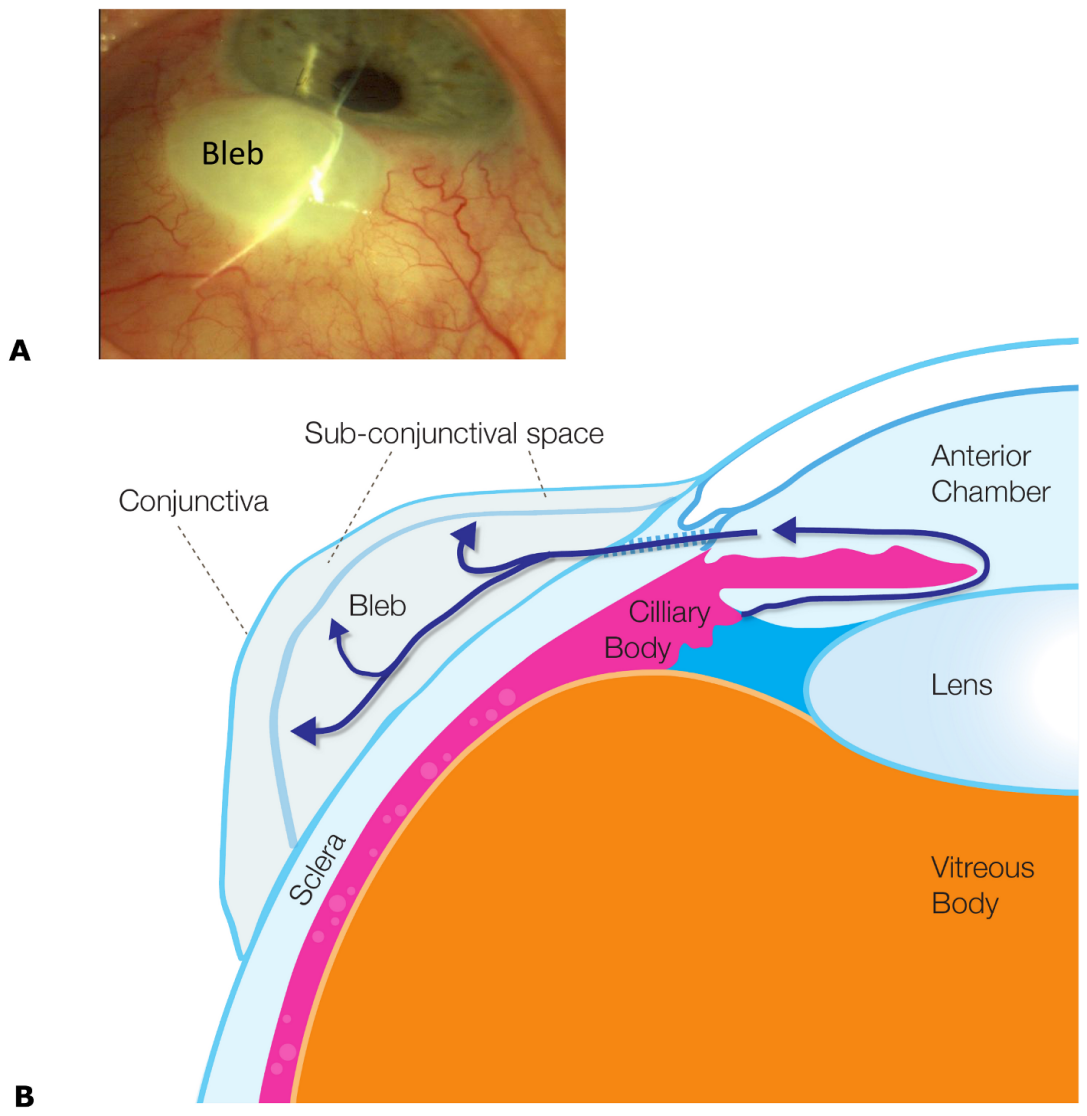
Anatomy of a bleb. (A) Photograph of an eye with a fluid bleb (labeled). Note in this case the lack of observable capillaries above the bleb. (B) Cross-sectional schematic of the key anatomical structures in an eye containing a bleb as a result of a trabeculectomy. Aqueous humor is produce by the ciliary body into the anterior chamber. The aqueous humor passes in front of the, lens through the iris and out through the sclera into the bleb. Note flow through the trabecular meshwork or via the uveoscleral pathway has not been depicted.

Glaucoma is the World's second leading cause of blindness, with advancing age and elevated intraocular pressure (IOP) being the major risk factors that predispose to optic nerve degeneration. Glaucoma surgery is the most effective means for lowering intraocular pressure typically to between 10 and 15 mmHg [1]. The most common surgical approach is to drain aqueous humor to surrounding eye tissue (sub-conjunctiva), where it can then be absorbed by capillaries (See Figure 1). The sub-conjunctiva is a thin layer of tissue between the sclera (a tough fibrous opaque tissue sometimes known as the white of the eye) and the overlying conjunctiva (a clear mucous membrane which extends to cover the underside of the eyelids to acts as a lubricating layer and an immune barrier). That is, a new avenue for fluid removal from the eye is created through the sclera and into a tissue not normally exposed to eye fluid pressure, fluid shear or tissue swelling. A fluid bleb generally forms in this tissue just outside the point of fluid drainage through the sclera from the anterior chamber of the eye. The fluid bleb is predominantly a fluid filled cavity formed when the tissue swells or is displaced by excess fluid accumulation, although the bleb may have some internal structure. Typically fibrous tissue (or scarring) occurs, which encapsulates the fluid bleb (and here is referred to as the bleb capsule) forming a scar layer. The geometry of this resulting fluid bleb is thought to be a significant determinant of the efficacy of fluid removal by the sub-conjunctiva, and so success of the glaucoma surgery. Consequently bleb grading systems have been developed to predict the long term success of an individual procedure [2]. Further, various glaucoma drainage implants have been developed in an effort to dictate bleb formation and assist fluid dispersal in the sub-conjunctival tissue [3]. Despite much being known of the inflammatory factors that contribute to increased subconjunctival scar formation, the physical principles linking bleb properties to stable intra-ocular pressure and surgical success have not be clearly elucidated, and implant design has mostly lacked a rational design foundation.

Failure of glaucoma surgery is a very significant health and surgical problem [4] and the need for a robust and cost effective means for improving surgical outcomes is clear. This is particularly true in many developing countries where, for logistic reasons, glaucoma surgery is the only viable therapeutic option. Unfortunately long-term success rates of glaucoma filtration surgery are generally poor. A recent review of 5 commonly used drainage implants reported that surgical failure occurred in ∼25% of cases [1], where failure is defined as the inability of the bleb or capsule around a drainage implant to accept or distribute the fluid presented to it without rupture additional scar tissue formation (that is, tissue remodeling is ongoing and tissue stability is not achieved). Bleb failure with scar formation is due to changes that occur in the tissue's ability to conduct fluid away from the source, as a result of collagen deposition and bleb capsule formation. A decrease in the hydraulic conductivity of the bleb capsule leads to a rise in fluid pressure within the bleb (and so within the aqueous humor of the eye). This rise in pressure and the continual flow of fluid from the bleb presumably changes the mechanical and chemical environment around the bleb. This abnormal tissue environment potentially leads to further tissue remodeling, or a vicious cycle (*i.e.* a positive feedback processes) towards bleb failure.

There are various physical mechanisms that may be postulated to contribute to this tissue remodeling and bleb failure. For example, excessive deformation or fluid pressure may lead to ischemia of the tissues (due to blood vessel collapse [5]) and a subsequent hypoxia and accompanying inflammatory response [6], while excessive fluid shear forces may result in changes in extracellular matrix (*e.g.* collagen density and alignment produced by fibroblasts [7], [8], [9].

The main aim of this paper is to begin to quantitatively define the factors affecting the fluid distribution and removal in and around a bleb. The model developed will focus on the relationship between bleb size and shape, tissue hydraulic conductivity, sub-conjunctival tissue fluid absorptive capacity, outflow facility, interstitial fluid pressure and IOP.

To date, the hydraulic forces acting on blebs have not been quantified. These forces acting within and around a bleb may be estimated using the theory of porous media [10], [11], [12]. Most biological tissues may, in an abstract sense, be conceived as a fully-saturated two-phase (*i.e.* liquid and solid phases) porous media. The porous media is made up of extracellular and intracellular fluid (the liquid phase), and extracellular matrix and cell cytoskeleton (the solid phase). The main advantage of the porous media model is that it can describe the interaction of a fluid moving through a porous structure without focusing on the specific, often heterogeneous, microstructure of the tissue. Instead up-scaled or averaged parameters are used to describe this interaction. The theory of flow through porous media, or more elaborate theories such as the theory of poroelasticity, may be employed to estimate the forces acting in tissues. Possible forces that may be important in the mechanical environment sensed by cells include (i) the stretch of solid matrix in the porous media (ii) an increase in the hydrostatic fluid pressure in the bleb and surrounding tissues, and (iii) fluid shear forces within the porous medium. In this paper, we focus on the later two forces, and use the theory of flow through porous media to estimate these quantities.

## Methods

The computational model used to describe fluid production and removal in the eye and surrounding tissues, including into the fluid bleb and sub-conjunctiva, is now described. This model integrates known key processes (see Figure 1B) and includes: fluid production by the ciliary body, fluid removal from the eye via the trabecular meshwork and uveoscleral outflow routes (in both normal and diseased states), fluid removal from the eye via the glaucoma drainage device and a fluid movement into and sorption by the sub-conjunctival tissues. Specific details of how each of the model elements are defined and incorporated into the model are discussed below.

The basis of our model is presented in two parts. First is the fluid production and loss in the eye. This will be followed by a description of the sub-conjunctival tissue containing a fluid bleb. The two models for each tissue are connected via a flux boundary condition.

### Fluid production and loss in the eye

In the eye fluid is produced by the ciliary body. It then passes between the iris and the lens and exits via either the trabecular mesh into Schlemm's Canal, or via the so-called uveosceleral pathway at the back of the eye. Thus the fluid in the eye is continually replenished by the cilary body and removed through the trabecular meshwork or uveoscleral pathway. A restriction in the outflow through the trabecular mesh (*i.e.* reduction in the trabecular outflow facility) is thought to be the primary cause of elevated IOP, although an increase in ciliary production or a decrease in the uveosceleral removal would also lead to elevated IOP. Consequently surgical interventions to decrease IOP attempt to increase outflow through the trabecular mesh, or provide an additional (new) fluid outflow pathway in the form of a hole through the sclera to drain into the sub-conjunctival tissue (the focus of the model developed here).

The change in fluid volume in the anterior chamber of the eye can be described using conservation principles. That is the time rate of change of the anterior chamber volume (V) is
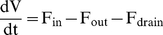
(1)where; 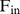 represents the total rate of production of fluid volume by the ciliary body, 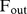 represents the rate of fluid volume loss from the eye via the trabecular meshwork and uveoscleral route and 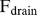 represents the rate of fluid volume loss from the eye via the surgical pathway into the sub-conjunctival tissue. Note we have assumed an incompressible fluid to obtain Equation (1).

Throughout this paper it is assumed that the fluid production rate 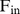 by the ciliary body, is constant at 2.5 µLmin^−1^ and is independent of IOP, although it is known that there is some daily variation (1.5–3 µLmin^−1^ reported in [13]). This assumption can be later relaxed to account for feedback with IOP (pseudofacility) or the effects of medication.

Following the Goldmann equation [14] it is assumed that F_out_ is described by
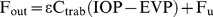
(2)where F_u_ is the outflow via the uveoscleral pathway, EVP is the episcleral venous pressure. Representing a minor departure from the Goldmann equation C_trab_ is defined here as the facility of outflow via the trabecular pathway for a *healthy* eye and the parameter ε has been introduced to allow us to vary the effective outflow facility to model diseased eyes. That is, when modeling healthy eyes, ε = 1, and when modeling glaucoma, where it is thought that there is a reduction in the trabecular outflow facility, ε can take the value 0<ε<1.

Prior to surgery, where there is no surgical pathway, F_out_ = F_in_ and if F_out_, C_trab_, IOP and EVP are known (*e.g.* from the literature, see Table 1), the ‘typical’ flow through the uveoscleral pathway F_u_ can be estimated from equation (2). In the more general case following surgery, for simplicity we assume here that F_u_ does not vary with IOP, or between healthy eyes or those with glaucoma, and is 15% of F_in_ [13], [15], [16].

**Table 1:** Eye model parameters used in this study to describe the aqueous humor production and loss in the eye.

Parameter	Description	Assumed value	Reference
F_in_	Rate of production of aqueous humor	4×10^−11^ m^3^s^−1^ (2.5 µL min^−1^)	[13]
F_u_	Rate or fluid outflow through the uveoscleral pathway	6.25×10^−12^ m^3^s^−1^ (0.15F_in_)	[13], [15], [16]
C_trab_	Trabecular facility of outflow for healthy eye	3.8×10^−14^ m^3^ s^−1^ Pa^−1^ (0.3µLmin^−1^mmHg^−1^)	[36]
K_r_	Mean (ocular) rigidity coefficient	1.7×10^9^ Pa m^−3^ (0.0126 mmHg µL^−1^)	[16]
EVP	Episcleral venous pressure	1.3×10^3^ Pa (10 mmHg)	9 mmHg [37]; 9.5mmHg for control and 11.6–12.1 mmHg for glaucoma [38]
R	Radius of drainage tube	1×10^−4^ m	Molteno3 internal radius 1.7×10^−4^ m [26]
L	Length of drainage tube	1×10^−3^ m	Sclera thickness 0.5–1×10^−3^ m [39]; Molteno tube length 0.75–18×10^−3^ m [26], [40]
μ	Viscosity of aqueous humor at 37C	7×10^−4^ Pa s	Viscosity of water at 37C is 7×10^−4^ Pa s [41]
ε	Fraction of normal outflow facility	0.1	See comments in results and Figure 4

It is assumed that aqueous humor flow to the sub-conjunctival tissue is via a cylindrical tube or hole and so can be described using the well-known Hagen-Poiseuille equation. That is
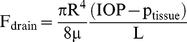
(3)where R and L are the radius and length of the tube, respectively, μ is the aqueous humor viscosity and p_tissue_ is the fluid pressure in the bleb/tissue immediately next to the outlet of the drainage tube.

The change in volume given by Equation (1) can be related to the change in IOP using an empirical linear relationship obtained by [17] for living human eyes by injection 200 µL saline solution into the eye at ∼270 µL/min and observing an increase in IOP from 10–55 mmHg. This linear relationship can be expressed as:
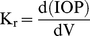
(4)where K_r_ is called the mean (ocular) rigidity coefficient. Note (4) is asymptotically correct for sufficiently small changes in pressure and volume. Equations (1)–(4) can now be combined to derive a rate form of the Goldmann equation generalized here to include fluid loss to the sub-conjunctival tissue.
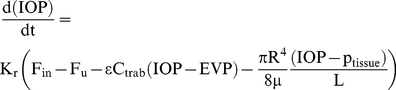
(5)Equation (5) provides a link between IOP and the fluid volumetric flow rate entering the sub-conjunctival tissue (and the fluid hydrostatic pressure at which it enters). During each simulation 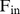, 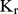, 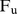, 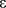, 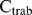, 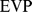, 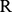, 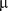 and 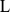 are held constant, with values shown in Table 1 unless stated otherwise, and Equation (5) is solved for IOP and p_tissue_, once a model for fluid transport into the tissue is introduced below. This Equation provides the boundary condition required to link the eye model to the tissue model to be described below.

### Fluid transport and removal in the sub-conjunctival tissue

The surgical pathway of exit of the aqueous humor assumed in the model is as follows. The fluid leaves the eye via the surgical pathway (described by Equation (3)) and enters the bleb. From there the fluid moves out into the surrounding sub-conjunctival tissue and is absorbed into sub-conjunctival capillaries. The sclera and conjunctiva are assumed to act as a fluid barrier, as a bleb is formed between these two layers.

A porous media model will be used to describe the movement of fluid into a bleb and through the sub-conjunctival tissue and its removal (or production) by the microvasculature. Porous media models have been used previously to model a wide range of other tissues [12], [18]. In this approach it can be assumed that fine details of the microvasculature (*i.e.* its branched architecture, distribution of vessels sizes *etc*) and tissue do not need to be modeled explicitly. Instead fluid movement through the bleb and sub-conjunctival tissue can be described using Darcy's law and a distributed fluid sink. Darcy's law is stated mathematically as,
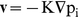
(6)where **v** is the homogenised fluid flux per unit area. Note v is a vector with units of ms^−1^ and is often referred to as the Darcy velocity. p_i_ is the hydrostatic pressure in the tissue interstitium (units Pa), K is the hydraulic conductivity (units of m^2^s^−1^Pa^−1^) of the tissue and represents the ratio of the intrinsic conductivity of the tissue pore space geometry and the fluid viscosity. The term 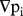 describes the gradient of pressure. Darcy's Law is a constitutive relation and expresses the finding that fluid movement through a porous medium is a linear relationship between the fluid flow and the gradient of the fluid pressure. The hydraulic conductivity is the proportionality constant in Darcy's Law. We note here that the hydraulic conductivity may vary spatially to represent the properties of different tissues (*e.g.* scar and sub-conjunctiva). Potentially the hydraulic conductivity may change over time, due to tissue remodeling around the bleb in response to say, fluid pressure.

Assuming an incompressible fluid phase and a static solid phase the porous media mass conservation equation for the fluid phase is
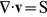
(7)Equation (7) can be combined with (6) to give the governing equation for fluid transport in a tissue,
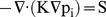
(8)Here S accounts for the volume of fluid produced or removed per unit time by capillaries in a unit volume of tissue and takes on a positive value for fluid production and a negative value for fluid removal. The term S can be described using Starling's law [18]

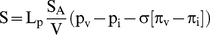
(9)where L_p_ is the hydraulic permeability of the vessel wall (units of ms^−1^Pa^−1^), S_A_/V is the surface area of blood vessel walls per volume of tissue (units m^−1^), p_v_ is the microvasculature pressure (in Pa), 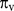 and 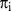 are the microvasculature and interstitium oncotic pressure respectively (units Pa) and σ is the reflection coefficient. Note steady state equations have been derived, without an additional governing equation to describe tissue swelling. This was done because the transient processes such as tissue deformation (*e.g.* due to swelling) occur much faster than the timescales of interest (*i.e.* times consistent with long term stable IOP). The changes in tissue properties due to swelling can still be addressed by assuming the final bleb-tissue geometry and adjusting the hydraulic conductivity accordingly. Recall the focus of this paper is to investigate the efficiency of fluid removal by a bleb given a specific bleb size and surrounding tissue properties. The derived equations are consistent with this aim.

The unknown parameters L_p_, S_A_/V, 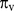, 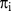, σ and p_v_ are estimated using the work of Jain *et al.* [18], who found these parameters for other tissues, including muscle and carcinomas. As shown in Table 2 Jain *et al.*'s [18] review has been used to inform the current study's choice of L_p_, S_A_/V, 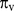, 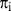, σ and p_v_. A difference between normal tissue and tumors was reported for L_p_, with tumor vessels more permeable. We have chosen a value for L_p_ near that of tumors, as in early bleb formation the tissue is undergoing swelling, large deformation and acute inflammatory response, which typically result in leaky vessels [19], [20], [21], [22]. That is, in the few weeks post surgery inflammation would likely increase vasculature permeability. Other relevant factors known to increase vasculature permeability are hypoxia [20] and fluid shear stress [21].

**Table 2:** Parameter estimation for model of fluid transport in sub-conjunctival tissue.

Parameter	Value	Value used
Lp hydraulic permeability of blood vessel wall	Normal: 2.7×10^−12^ ms^−1^Pa^−1^ (3.6×10^−8^ cm s^−1^ mmHg^−1^) Tumour: 1.4×10^−10^ ms^−1^Pa^−1^ (1.86×10^−6^ cm s^−1^ mmHg^−1^) [8], [18], [23]	1×10^−10^ m s^−1^Pa^−1^
K hydraulic conductivity	1–400×10^−14^ m^2^s^−1^Pa^−1^ [8], [18], [23]	Sub-conjunctival tissue: 50×10^−14^ m^2^s^−1^Pa^−1^, Bleb: 50,000×10^−14^ m^2^s^−1^Pa^−1^, Scar tissue: 5×10^−14^ m^2^s^−1^Pa^−1^
S_A_/V vessel wall area per tissue volume	5×10^3^–25×10^3^ m^−1^ (50–250 cm^−1^) [18]	Sub-conjunctival tissue: 10×10^3^ m^−1^, Bleb: 0 m^−1^, Scar tissue: 10×10^3^ m^−1^
p_v_ vasculature pressure	1.3×10^3^–3.3×10^3^ Pa (10 mmHg) [18]	1.3×10^3^ Pa (10 mmHg)
π_v_ vessel oncotic pressure	2.6×10^3^ Pa (20 mmHg) [18]	2.6×10^3^ Pa (20 mmHg)
π_i_ interstitium oncotic pressure	1.3×10^3^ Pa (10 mmHg) [18]	1.3×10^3^ Pa (10 mmHg)
σ reflection coefficient	0.91 [18]	1

The parameter values listed will be used throughout the study unless explicitly stated otherwise (*e.g.* when performing parametric study).

Jain *et al.* [18] also report a range for S_A_/V of 50–250 cm^−1^ which is invariant between normal and tumor tissue. We have chosen an intermediate value towards the lower end, specifically 100 cm^−1^ as there is qualitatively fewer vessels in the sub-conjunctival tissue than might occur elsewhere. Further, it is assumed that the surface area of vessels to bleb volume is negligible *i.e.* blebs contain few vessels. The scar tissue is assumed to maintain the same S_A_/V as sub-conjunctival tissue.

Swartz and Fleury [8] and Levick [23] each compiled a list of values of K in a range of tissues. They found that K varies from ∼1×10^−14^ m^2^s^−1^Pa^−1^ for sclera and corneal stroma up to 400×10^−14^ m^2^s^−1^Pa^−1^ for the vitreous body. The mesentery has a hydraulic conductivity of 30–200×10^−14^ m^2^s^−1^Pa^−1^. By comparison [18] obtained an average hydraulic conductivity for a wide range of tissues and tumors as 20×10^−14^ m^2^s^−1^Pa^−1^. Further the hydraulic conductivity can increase dramatically when the tissue becomes hydrated and swells [8], [13], [23], [24]. A first estimate of the hydraulic conductivity of the sub-conjunctival tissue is assumed to be the intermediate value of 50×10^−14^ m^2^s^−1^Pa^−1^. Due to the extensive hydration of the bleb itself we assume that it offers little resistance to fluid flow and so give it a hydraulic conductivity of 50,000×10^−14^ m^2^s^−1^Pa^−1^ (*i.e.* 1000 times that in the sub-conjunctival tissue) [25]. As we shall show later, this high value is sufficient for the hydrostatic pressure to be effectively uniform throughout the bleb. In simulations involving scar tissue we choose a hydraulic conductivity that is low but still higher than that of the sclera. Specifically for the scar tissue we use K = 5×10^−14^ m^2^s^−1^Pa^−1^ (or 10% of the hydraulic conductivity assumed for the sub-conjunctival tissue).

Although parameter values described above are best estimates, a parametric study is also performed to assess how results would be modified for a different choice of parameters.

**Figure 2:**
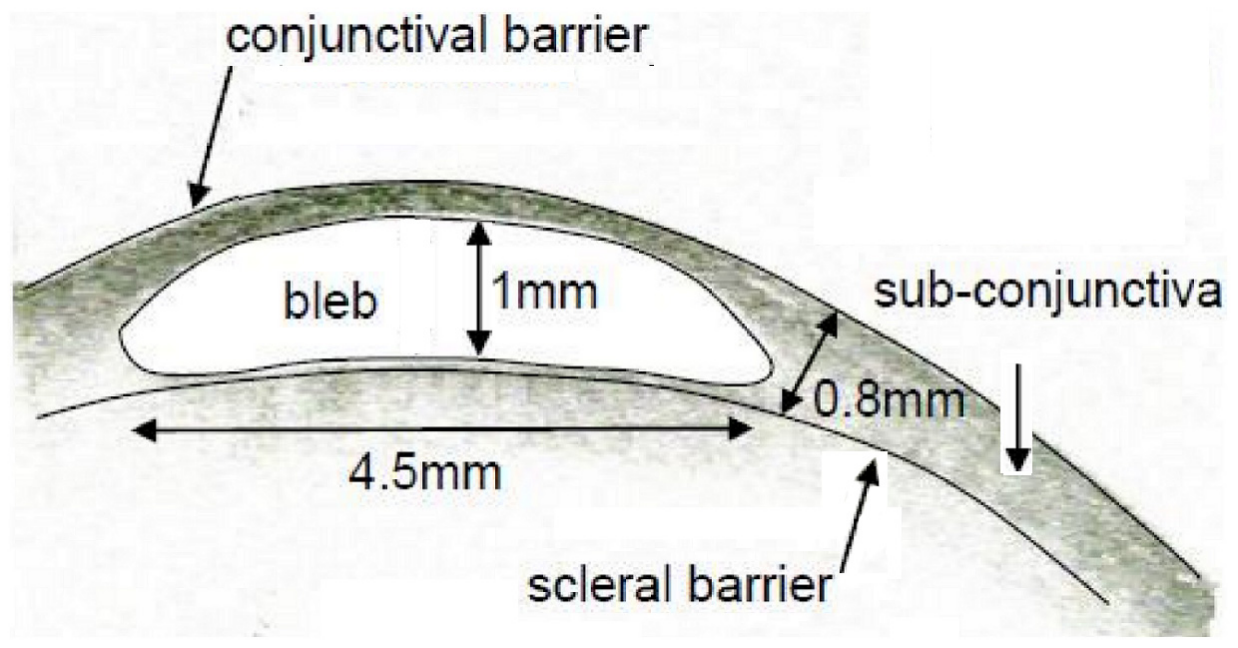
Bleb geometry. Cross-sectional Ocular Coherence Tomography image left eye of a simple bleb morphology, functioning bleb of IOP 10 (“Visante OCT” Zeiss HE 10/1/37 RVEEH – image date 8/4/08). Note this does not imply that this bleb size or shape is optimal. Dimensions shown are the approximate dimensions of the bleb to be used as a standard for model construction. Note it is unclear where the sclera barrier is from this image and so the line drawn is approximate. In the subsequent models (*e.g.* see Figure 3) we assume the bleb sits directly on the sclera and is axisymmetric. Further we assume the curvature of the sclera is unimportant to the mass transport problem.

**Figure 3:**
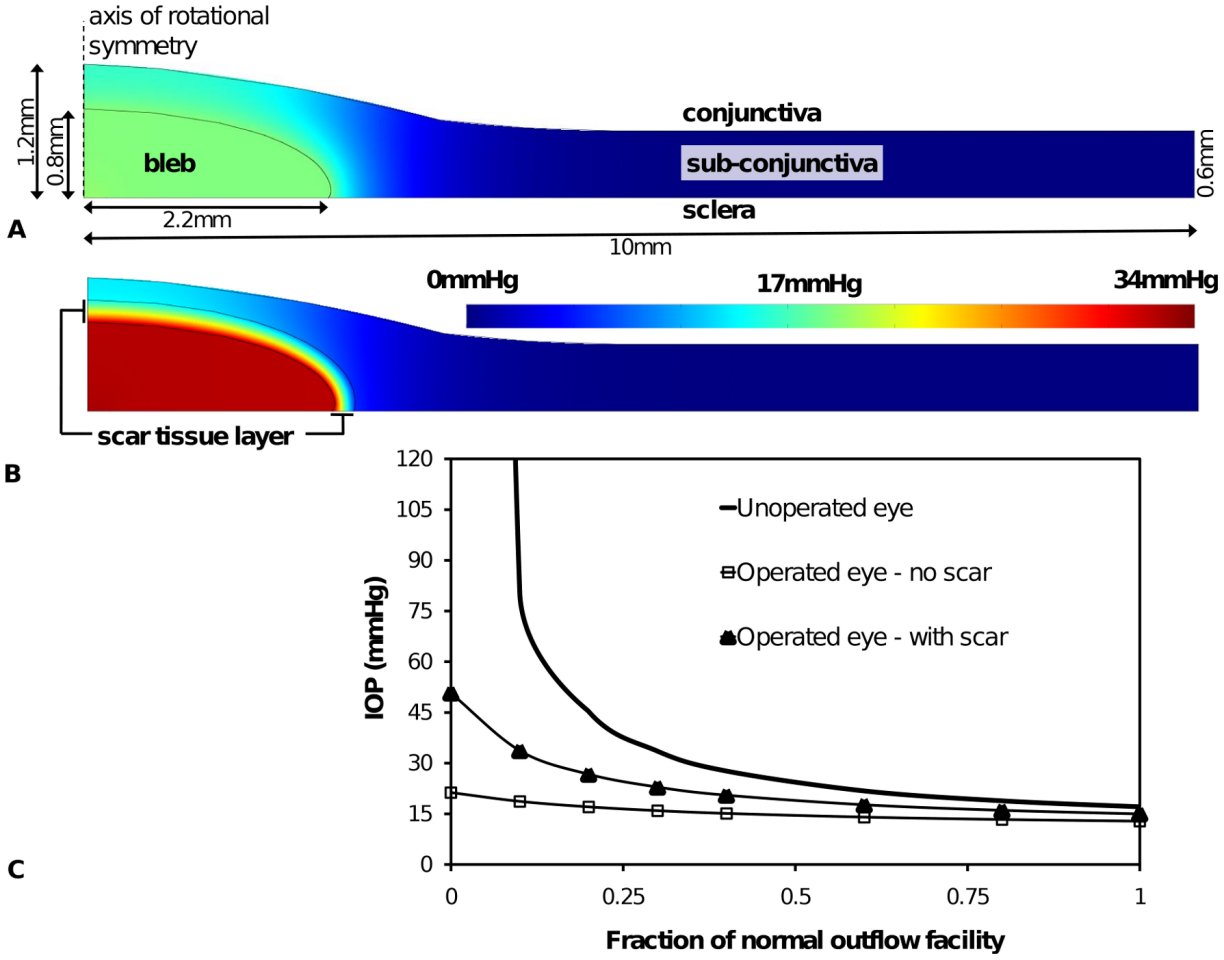
Geometry and pressure predictions of the standard functioning bleb. (A) Without scar tissue and (B) with a layer of scar tissue surrounding bleb. Color scale indicates interstitial fluid pressure. (C) IOP with degree of blockage ε of the fluid flow through the trabecular meshwork leading to a reduced outflow facility. Three cases are shown. The first (solid line) shows increase in IOP with reduction of outflow facility for an unoperated eye. The second case (solid line with squares in (C)) shows the relatively controlled IOP due to additional outflow pathway of 100µm radius into a typical bleb within normal sub-conjunctival tissue. The third case (solid line with triangles) shows the effect of a scar layer of uniform thickness (0.2mm) encapsulating the bleb.

Various bleb and tissue dimensions used in the model are based on the functional ‘standard’ bleb shown in Figure 2. Specifically, an idealized axisymmetric model is adopted in which we consider a circular region of the sub-conjunctival tissue centered on a bleb, which is depicted in Figures 3A (no scar tissue) and 3B (with scar tissue). The inner surface of the tissue (which is in contact with the sclera) is treated as a flat circle of radius 10 mm, and the sub-conjunctiva has normal thickness 0.6 mm. The bleb occupies an axisymmetric region in the middle as shown in the Figure 3. The standard bleb has a radius of 2.2 mm and maximum height 0.8 mm. A layer of scar tissue of thickness 0.2 mm optionally covers the surface of the bleb. To accommodate the height of the bleb, the thickness of the sub-conjunctival tissue increases smoothly to a central maximum of 1.2 mm. Continuity of flux and pressure are assumed on the internal boundaries. A no flux boundary condition is applied on all the external boundaries of the sub-conjunctival tissue and on the bottom surface (in contact with the sclera) of the bleb, except for a central circle of radius 100µm at the bottom surface of the bleb. At this central surface of the bleb fluid drains into the bleb from the anterior chamber via a 100µm radius tube, and so the pressure is *p*_tissue_ and the total flux is set at *F*_drain_, with the flux assumed uniform over the boundary (also implying a uniform interstitial fluid velocity across this boundary). The system is solved in two dimensions and regularity conditions are imposed on the axis.

The model is solved numerically in the commercial FEM solver COMSOL Multiphysics. When solving Equations (5) and (8) in COMSOL two domains (or geometries) are used. One domain, represents the bleb and sub-conjunctival tissue, is used to solve the 2D axisymmetric PDE problem (Equation (8)). The other domain is used to solve the fluid mass balance ODE problem in the eye *i.e.* Equation (5). In COMSOL variables can be passed between these two domains. In our case we pass p_tissue_ back to the ‘eye domain’ to determine IOP, and pass F_drain_ (which can be calculated from IOP) to the ‘tissue domain’ to provide a tissue flux boundary condition at the bottom centre of the bleb. The default solver settings are used except for higher tolerance settings (relative tolerance: 10^−5^; absolute tolerance: 10^−6^). Approximately 40,000 triangular mesh elements were used.

## Results

In Figure 3 model predictions of the interstitial pressure distribution in the sub-conjunctival tissue are shown for two scenarios: a bleb surrounded by normal tissue (Figure 3A) and a bleb encapsulated by a thin layer (0.2mm) of scar tissue and surrounded by normal tissue (Figure 3B). In each case the functioning bleb geometry depicted in Figure 2 has been used. It is seen that scar tissue was predicted to lead to a higher bleb pressure (and IOP). In this example bleb pressure increased from 17mmHg to 32mmHg when a scar was present. Further, if no scar was present approximately 75% of the eye fluid production passed through the bleb to be absorbed in the sub-conjunctival tissue, whereas a scar layer reduced the volume of fluid passing through to the bleb to 55% of the eye fluid production. That is, fluid filtration by the bleb was less effective once a scar developed, with the consequence of elevated IOP. As IOP increased with scar formation, proportionally more fluid was forced to exit via the trabecular pathway, as the pressure difference across the trabecular meshwork increased. This was a clear demonstration of scar formation leading to increased IOP (and so failure of the glaucoma surgery).

On the other hand scar formation was seen to reduce the sub-conjunctival tissue's exposure to interstitial pressure. The highest tissue pressure was predicted to occur directly above the bleb, whether or not a scar was present. If scar formation is in response to elevated pressure, either directly or indirectly (through ischemia induced by capillary occlusion), our model would suggest that a scar would form first above the bleb and that this scar would limit the tissue's exposure to the adverse fluid pressure, however the subsequent increase in IOP would adversely affect retinal tissue. It has been reported that uncontrolled IOP tends to be associated with thick, vascularized bleb walls [4]. These thick, vascularized walls could be the result of scar formation and angiogenesis in response to tissue hypoxia induced by high fluid pressures.

According to Equation (9), fluid absorption has a linear relationship with the interstitial fluid pressure. It can be estimated from the distribution of interstitial fluid pressure shown in Figures 3A and 3B that the majority of the fluid absorption by the sub-conjunctival tissue occurs within ∼1mm of the bleb (for the case of no scar), as the interstitial pressure has approached 0mmHg within this range. When a scar develops, absorption occurs in an even smaller region of tissue within ∼0.1mm from the bleb or rather within the scar itself.

Figure 3C contains model predictions of filtration bleb performance in reducing IOP, in comparison to an unoperated eye and the two blebs depicted in Figures 3A and 3B (*i.e.* standard bleb with and without a scar layer). In this figure IOP is shown for a range of failing outflow facility (described by a decrease in model parameter ε). Operative induced increases in facility (surgical facility) can lead to a substantial reduction in IOP, with the effect on IOP more obvious with a reduction in the residual outflow. When the trabecular pathway was completely blocked (ε = 0), the model predicted an IOP of 21mmHg for the operated eye, compared with a very high pressure in the unoperated eye (actually a steady state IOP cannot be achieved). (Note the predicted continuous increase in pressures in the unoperated eye when ε = 0 is not biologically possible, as ischemia of the ciliary processes would restrict inflow). The addition of a layer of scar tissue was seen to increase the IOP for all ε toward the case of an unoperated eye. This demonstrates that scar tissue reduces the ability of a bleb to control IOP. This result is consistent with the clinical experience of glaucoma surgery.

Although not shown, the radius of the tube connecting the eye from the bleb was varied between 20–400µm, and the tube length was varied between 1–20mm without significant effect on the results contained in Figure 3. This indicates that the tube offers very little resistance to fluid flow in comparison to the sub-conjunctival tissue or trabecular mesh, at least within this range of tube radii or length. Consequently the fluid pressure inside the bleb was effectively the same as the IOP, to within 1 mmHg, and in all following results bleb pressure is used as a measure of IOP. The radius of the tube (or ostium) typically used in glaucoma surgery is significantly larger than the minimum required (*e.g.* 340 µm internal diameter used in the Molteno tube [26]), and it suggests that, based on fluid flow requirements alone, significant miniaturization of the tube could occur. There is the potential for the drainage tube to collapse or kink, which would introduce a larger pressure difference between the IOP and the bleb, and reduce the effectiveness of the bleb in distributing fluid to the sub-conjunctiva.

**Figure 4:**
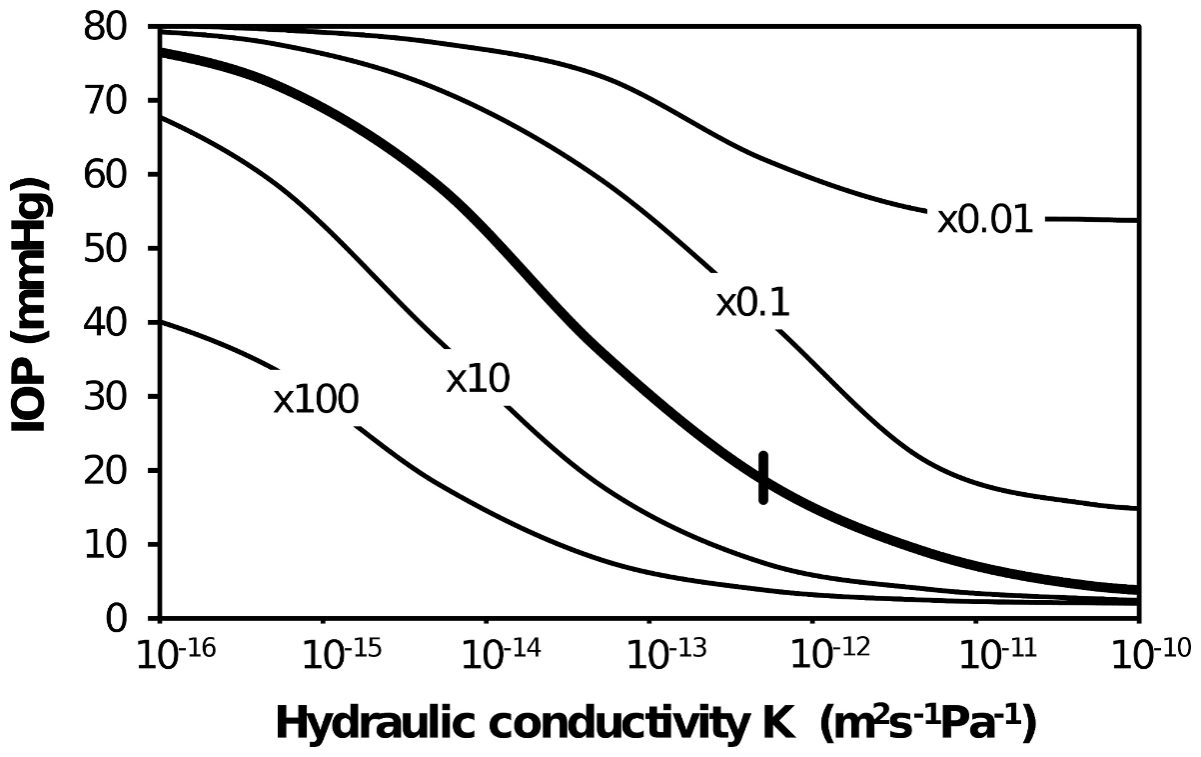
Variation of IOP with tissue properties for the standard bleb shown in **Figure 2** and **3A**. Tissue properties varied are tissue hydraulic conductivity K (bleb K kept constant) and L_p_S_A_/V (a measure of capacity of tissue to absorb fluid). A scar layer has not been included. Heavy solid line represents best estimate of absorption capacity over a range of K. Other solid lines represent variation in IOP with K for L_p_S_A_/V changes by ×100, ×10, ×0.1, ×0.01 from original L_p_S_A_/V. Short heavy vertical line indicates best estimate of K and L_p_S_A_/V. *i.e.* intersection of heavy solid and light dashed give best estimate of K and absorption capacity from Table 2.

Figure 4 shows results of a parametric study to examine how the predicted IOP varies for a change in the tissue properties of hydraulic conductivity and fluid absorption capacity (via an increase in capillary area within tissue and/or an increase in capillary wall permeability). It can be seen that an increase in hydraulic conductivity or an increase in absorption capacity generally led to a lower IOP. An increase in hydraulic conductivity lowers IOP because fluid can move further from the bleb and so involve more tissue in the removal of fluid. The downside of a higher hydraulic conductivity is that more tissue is exposed to higher hydrostatic pressures (not shown). Alternatively, an increase in fluid absorption lowered IOP, presumably by removing fluid more efficiently.

**Figure 5:**
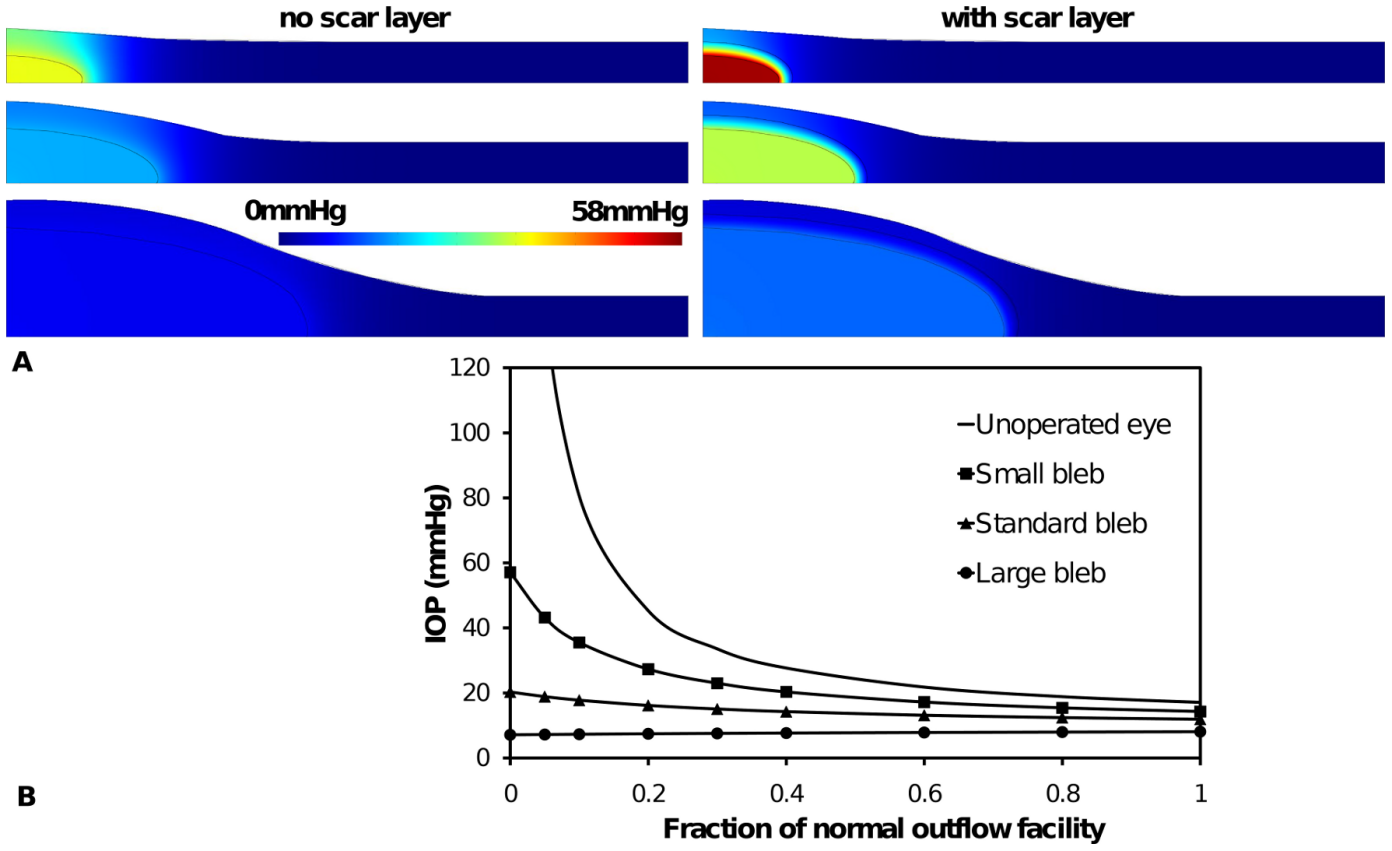
The effect of bleb size on aqueous humor interstitial pressure and IOP. (A) interstitial pressure distribution around three bleb sizes: a small bleb (top) 50% radius and height of functioning (standard) bleb (middle) and a large bleb (bottom) 200% radius and height of functioning bleb. Shown for each bleb size are the case of a scar layer and no scar layer. (B). The effect of bleb size on IOP for a range of outflow facility for the three blebs sizes shown in (A) without a scar layer.

**Figure 6:**
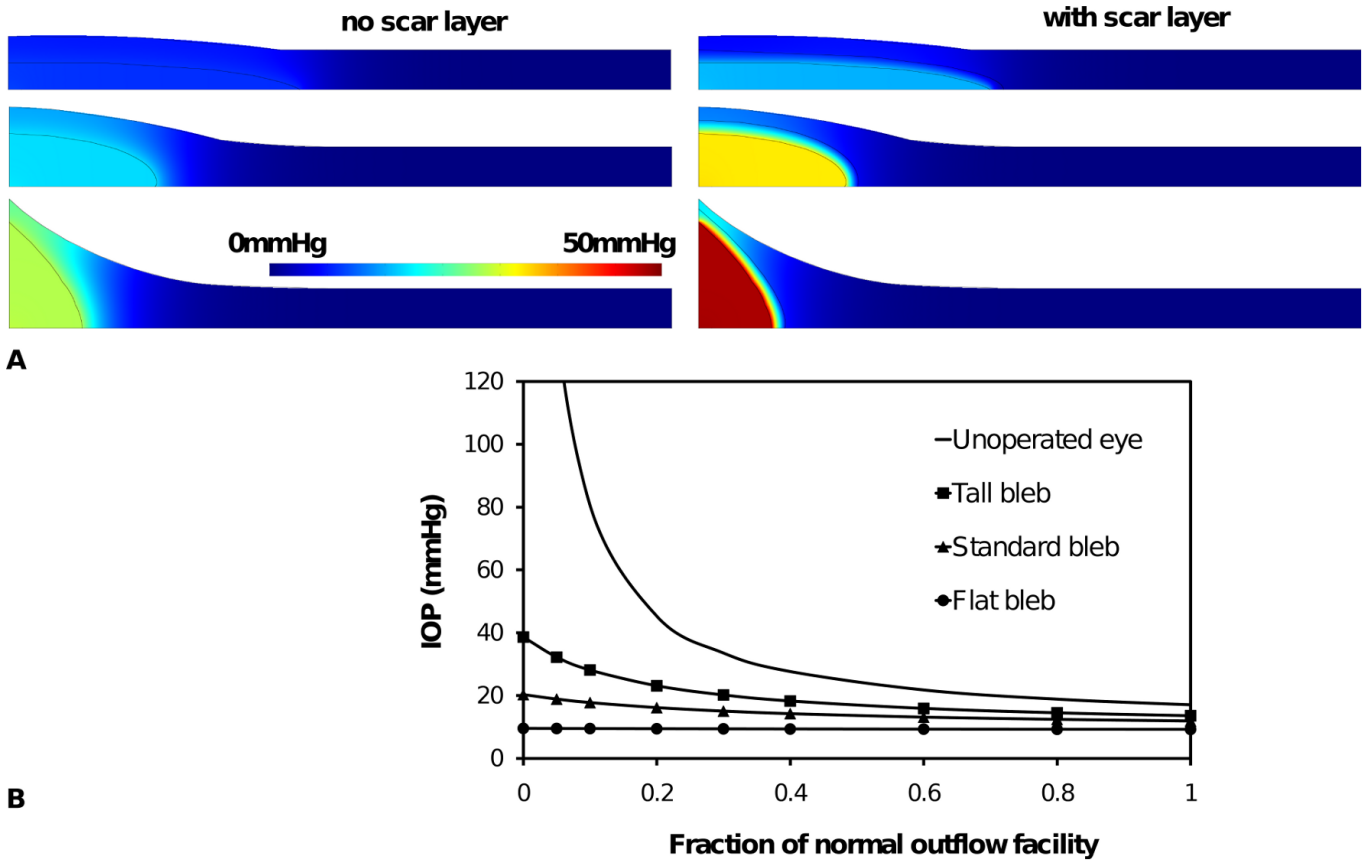
The effect of bleb aspect ratio on aqueous humor interstitial pressure and IOP. (A) Interstitial pressure distribution around three bleb shapes: a tall narrow bleb (top) with 50% radius and 200% height of standard bleb (middle) and a wide flat bleb (bottom) 200% radius and 50% height of standard bleb. Shown for each bleb shape are the case of a scar layer and no scar layer. (B). The effect of bleb aspect ratio on IOP for a range of outflow facility for the three blebs shapes shown in (A) without a scar layer.

From Figures 3 and 4 we surmise that changes in tissue fluid transport properties due to scar formation and vascularization are clearly important to IOP control. In Figures 5 and 6 we can conclude that the geometry of the bleb is also critically important.

In Figure 5 height and width of the functioning bleb were both either doubled or halved, so that the IOP and tissue pressure could be compared in the context of bleb size. That is, bleb volume was decreased or increased by a factor of eight and the bleb surface area was decreased or increased by a factor of four. It is immediately clear that a small bleb leads to higher IOP and tissue pressure. Again pressure was highest directly above the bleb. This accords with the clinical observation that small blebs often have areas of pallor overlying them, consistent with a reduction in capillary flow secondary to high tissue hydrostatic pressure. Total fluid absorption is directly related to the volume of tissue involved in the absorption. In small blebs, the fluid must travel further from the bleb before it can be completely absorbed. This fluid movement is limited by the hydraulic conductivity. From Figure 5B a larger bleb was predicted to have a controlled IOP, but at too low a level. Indeed hypotony is a significant risk of glaucoma surgery, and is mostly associated with a lack of definable boundaries to the bleb, thus the bleb is seen to ‘run away’ (*i.e.* an uncontrolled increase in size of the bleb). In all cases, inclusion of a scar layer elevates bleb pressure (and IOP).

In Figure 6 the height and width of the standard bleb are both changed by a factor of two, but in opposite directions (halved versus doubled), to create blebs of different aspect ratios *i.e.* a tall narrow bleb and a wide flat bleb. The model predicted that tall blebs lead to higher bleb and tissue pressure and IOP, compared with the wide flat bleb. The flat blebs had a more controlled IOP over a wide range of trabecular outflow facility (Figure 6B), but as with large blebs, they may increase the risk of hypotony. In all cases, inclusion of a scar layer elevated bleb pressure (and IOP). The results of Figures 5 and 6 indicate that there is an optimum bleb size and shape, which would avoid excessive scar formation but keep IOP in an acceptable range.

**Figure 7:**
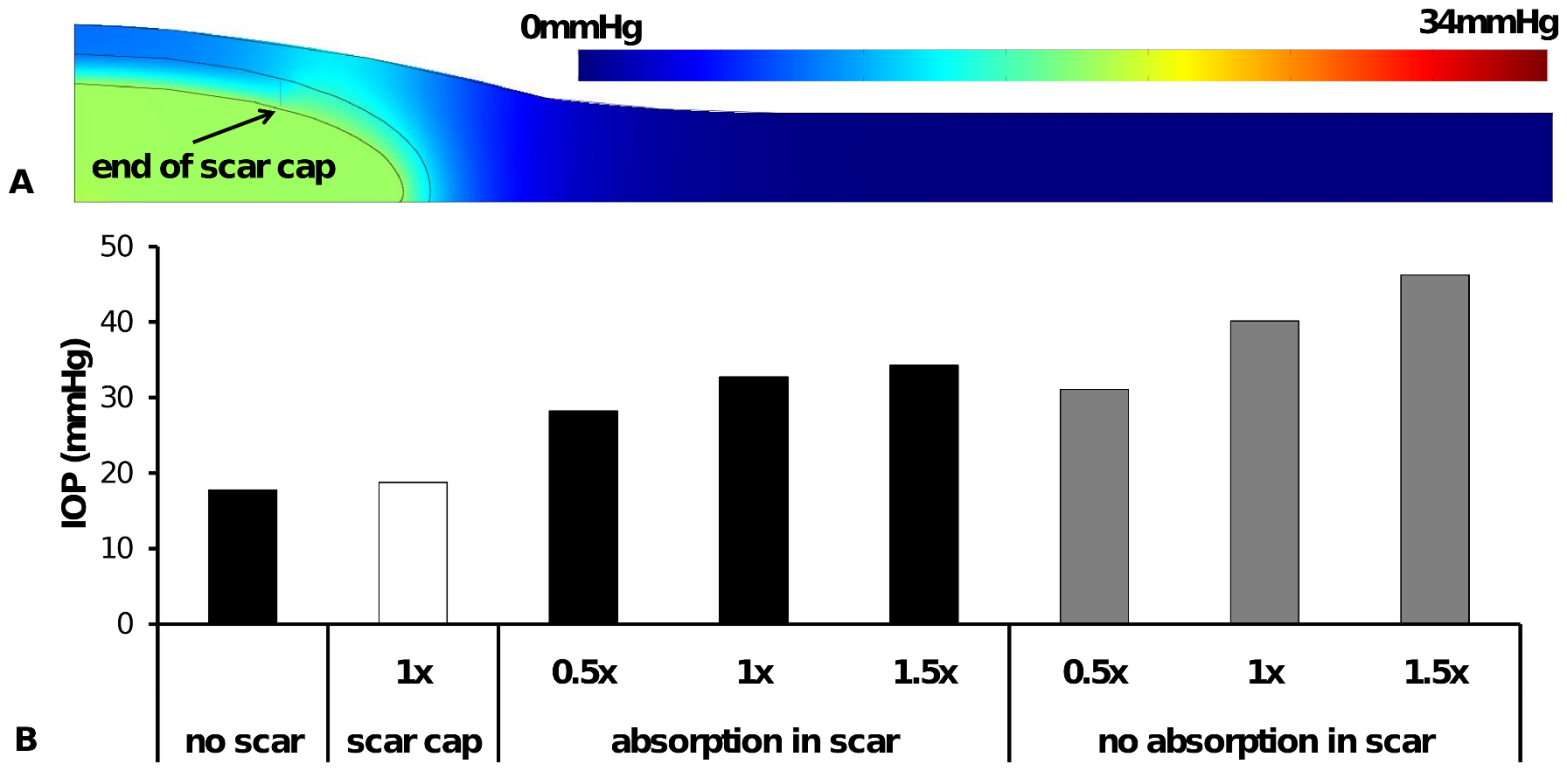
The effect of scar layer properties on aqueous humor interstitial pressure and IOP. (A) tissue pressure distribution around a bleb with a scar cap (partially formed scar layer) of radius 1.5 mm. Note pressure scale is same as that used in Figure 3A, (B) IOP for standard bleb without scar, the scar cap in Figure 7A and full scar layers of various thickness (1× refers to scar thickness used in previous figures *i.e.* 0.2mm; 0.5× refers to a scar layer of 0.1mm thickness and 1.5× refers to a scar layer of 0.3mm thickness). For each thickness of a full scar layer the effect of fluid absorption is also shown.

The results presented in Figures 3, 5 and 6 all suggested that prior to scar formation, the interstitial fluid pressure in the tissue is highest directly above the fluid bleb. Once a scar forms this tissue is somewhat protected from high pressures. Assuming scar formation is related to pressure we might expect scar to develop first at the top of a bleb. Figure 7A shows bleb and sub-conjunctival pressure distribution for the case of a scar cap of radius 1.5mm sitting above the bleb. The shielding of the tissue by the scar cap is clearly seen. Indeed the highest tissue pressure now occurs next to the outer edge of the scar cap. Presumably, if further remodeling were to occur, it would most likely happen at this outer edge, causing the scar cap to grow. On the other hand we see that the IOP is similar to that of a scar-free bleb, despite having a scar cap which is almost 70% the radius of the bleb itself (although it does constitutes a smaller component of the bleb surface area), and the sub-conjunctival tissue is exposed to lower pressures than the scar-free case. This suggests that it is the scar formation at the outer sides of the bleb which is most critical to maintaining a stable low IOP.

Figure 7B compares IOP for the cases of no scar, a scar cap, and a scar layer which completely encapsulates the bleb with one of three different scar layer thicknesses. Further, for the cases of completely encapsulating scars, the scar layer either contains capillaries (allowing fluid absorption within the scar layer) or excludes capillaries (no absorption within scar layer). In all cases no fluid absorption occurs within the fluid bleb. The three thicknesses presented are 0.2mm (*i.e.* the same thickness as used in previous figures – referred to as 1×), 0.1mm (*i.e.* 0.5× the thickness of standard scar layer) and 0.3mm (*i.e.* 1.5× the thickness of the standard scar layer). It can be seen, perhaps unexpectedly, scar thickness has only a minor effect on IOP when fluid is absorbed within the scar layer. Recall for Figure 3B it was noted that the majority of fluid absorption had already occurred within the scar layer, as the high interstitial pressure increased the absorption rate. If all the fluid is absorbed within the existing scar, increased fluid transport barrier offered by additional scar thickness becomes irrelevant, as the majority of fluid is absorbed before reaching this outer scar layer. If the scar layer is not able to absorb fluid (the case of no capillaries in the scar layer), the IOP is significantly elevated and there is a strong influence of scar thickness on IOP.

## Discussion

We have developed a model describing fluid flow in the eye after glaucoma surgery, where the factors influencing IOP, specifically aqueous humor production and outflow, bleb geometry, tissue conductivity and tissue absorptive capacity can be varied. The model accords with clinically derived experience in many key ways. Blebs have ‘successful’ characteristics which, although not exclusive, are generally described as ‘low’, ‘diffuse’ and ‘large without the formation of a distinct sub-conjunctival encapsulation’. This situation is described and explored in the model, which indicates the relationship between the size and internal structure and pressure relationships in and around the bleb. These model observations agree with clinical experience and published analyses of bleb morphology and function [27], [28], [29], [30], [31], [32].

A lowering of the hydraulic conductivity effectively seals off the fluid from leaving the bleb, such that it cannot be absorbed (recall Figure 3). In the extreme case, fluid cannot leave the bleb and the bleb is no longer an outflow pathway from the eye and the eye IOP is equivalent to that of an unoperated eye. Progressive scar formation is consistent with a lowering hydraulic conductivity. The uncontrolled IOP seen in thick, vascularized walled blebs [4] suggests that an increase in fluid removal efficiency by the increased vascularizaton is not enough to compensate for the decreased hydraulic conductivity of the collagen rich bleb wall. From Figure 4 we can estimate that a decrease in hydraulic conductivity or, alternatively, an increase in absorption capacity by an order or magnitude (or so) has similar effect on IOP. We therefore expect that the change in hydraulic conductivity due to scar formation is larger than the change in capillary area in the tissue. The implications of this model resonate clinically: that failure of IOP control is more directly related to decreases in hydraulic conductivity (*i.e.* due to scar formation) than to decreased absorption – *i.e.* the formation of relatively impermeable cap, rather than diffuse scarring and loss of absorptive capacity. In the history of glaucoma surgery research, histological thickness of the fibrous cap has been taken as a surrogate of surgical success [33], [34].

The success of glaucoma surgery rests is in the creation of an improved outflow facility from the eye; the key determinant of this being the hydraulic conductivity of the fibrous cap that develops around and delineates the bleb. Blebs with higher IOP tend to have thicker walls and therefore to be clearer in outline (*i.e.* not ‘diffuse’) and to have a smaller volume [32]. This phenomenon is clearly represented in the model, indicating that the more the resistance to flow through the bleb wall the higher the bleb pressure and the IOP.

It is interesting to note that the best estimate of the key model parameters of hydraulic conductivity and fluid absorption occur within the range of values in which the IOP is most sensitive to changes in these parameters – see Figure 4. This may provide a clue as to why there is a significant variation in surgical success in controlling IOP. That is, this model predicts small variations in tissue properties, as might be expected to occur within a population, will lead to considerable variation in the ability of a ‘typical’ bleb to control IOP, and a commensurate variation in surgical success. This variation is independent of any likely changes in tissue properties due to scarring and inflammation. If scarring were to occur, we would expect hydraulic conductivity to decrease and IOP to increase.

The current model does not include a pressure dependent change in the hydraulic permeability of the blood vessel walls L_p_. Although the exact mechanism is still in debate, there are now several studies that report the reduction in flow in capillaries subjected to external pressures above some threshold (*i.e.* a critical pressure) [5], [13], [35]. Exceeding the capillary closing pressure with tissue hydraulic pressure would cause the fluid absorption to dramatically decrease (*i.e.* a decrease in L_p_). Although the critical pressure at which capillaries close is not known for sub-conjunctival tissue, it has been reported that in retinal circulation blood flow is regulated for IOP up to 30mmHg, above 30mmHg blood flow decreases [13]. Assuming retinal capillaries are at a pressure 10mmHg above IOP, we may estimate the critical pressure as an upper bound of about 20mmHg. In this case we may expect the tissue above the small bleb in Figure 3, for example, to become ischemic, display a pale pallor and be the site of initial scar formation and lead to the so-called fibrous cap. That is, pallor of some portion of the bleb may, therefore, result from prolonged hydrostatic pressure causing capillary closure. Interestingly in the bleb shown in Figure 1, no red capillaries can be seen above the bleb, either they are beneath the bleb scar surface or blood is unable to pass through them due to capillary collapse. In the clinical situation pale blebs may also arise from chemical insults, such as the use of the mutagen Mitomycin-C at the time of the surgery. Pallor would seem to be a poor prognostic sign of the functional bleb as a certain level of capillarization is required for the absorption of aqueous. Pale blebs may also develop small leaks through the surface (which clinicians often called ‘ooze’) a drainage route that is not covered in this modeling, nor is it clinically desirable.

There are always limitations in our understanding of the issues that arise in computational modeling. For example, issues such as the closing pressure of the capillary bed in the bleb, the reduction in aqueous production of the eye at high IOP, the possibility of trans-conjunctival flow releasing aqueous from the absorption route and others. These limitations can be addressed in future studies, at least once more is known about the mechanisms behind these factors. However the ability of the current model to help understand the physical processes behind the clinically-observed bleb characteristics is an encouraging first step towards enabling a more informed glaucoma surgical protocol, a better bleb grading system, and more effective design of implants.

In conclusion, the model contained in this paper has helped illustrate the ‘balancing act’ required for successful fluid dispersal from a bleb following glaucoma surgery. Generally small, thick wall blebs are likely to be ineffective at allowing fluid to move into and be absorbed by tissue. These results suggest that small blebs expose tissue to high pressures, particularly directly above the bleb, inducing an ischemic tissue remodeling response, a reduction of hydraulic conductivity and an elevation of IOP. On the other hand, large blebs are less likely to undergo this remodeling process but may lead to hypotonic eyes. These results are consistent with bleb characteristics seen clinically.
